# Mummified Meat

**Published:** 1899-06

**Authors:** 


### MUMMIFIED MEAT.

THE mass of evidence accumulated during the recent inquiry into the question of the so-called embalmed beef shows very clearly that there was "something rotten in the State of Denmark." The characterization of the meat by officers and men varied from "unsuitable" to "putrid flesh canned with maggots in it."

The commissioners doubtless had the welfare of the troops very much at heart, but blind faith in bureaucratic routine made them derelict in their duty. All this hubbub over some tainted meat is likely to cause an indulgent smile on the face of the Confederate vet who in times agone accounted himself lucky if he possessed a handbreadth of "sow-belly" and a pocket full of corn, but now——well, things have changed since Willie died. W.

---

THE custom is very general among medical associations of inviting distinguished members of the medical profession from a distance to be present at their annual meetings and to participate in the scientific features by reading papers. It offers a prime attraction and draws a large number of members who come not only for the knowledge to be gained by listening to the subject as presented by a master, but also from motives of curiosity to behold him with the eyes of the flesh. From this point of view the custom is admirable and highly commendatory. But there are other features connected with it which somewhat detract from its expediency.

Respect for the man, interest in his subject and, above all, hospitality, unite in giving him license as to the length of the paper and the method of presenting it, in person or by proxy. But the effect upon the literary proceedings of the meeting is disastrous.

Members of the association who have come with carefully prepared papers to read, must yield their place on the program; even the business of the association is hurried to give place to the *pièce de résistance*. The result is that the order of the program is thrown hopelessly out of joint, no one knows when his paper is to be called, if indeed it be called at all, and one feels as though his right had been filched from him, and his disappointment is sharp and lasting.

To correct this and allow every member an opportunity of presenting his paper in its proper order, the program committee should set aside an hour say, for the presentation of the paper of the invited guest and for the lengthy discussion which usually follows. The presiding officer must under no consideration allow this time limit to be transcended. This plan would greatly facilitate the uniform advance in the proceedings, and courtesy and hospitality would be relieved of their strain.

It is a great pleasure to hear and see these distinguished co-workers and they are always most cordially welcome, but pleasure must be tempered with justice toward the members of the association, whose rights and privileges are entitled to the first consideration. W.

---

THE ninth annual meeting of the American Electro-Therapeutic Association will be held in Washington, D. C., on September 19th, 20th and 21st, 1899, under the presidency of Dr. F. B. Bishop, of Washington. Quite a number of papers of great Scientific value have been promised, and the Committee of Arrangements insures the members a very entertaining and pleasurable meeting. Aside from the sessions of the Association, the Committee has completed arrangements for a trip to Mt. Vernon, one to Arlington, and several other social features. The headquarters of the Association will be at Williard's Hotel, where special rates will be given to members and their families during the meeting.

